# Renoprotective effects and safety of sodium–glucose cotransporter 2 inhibitors in diabetic chronic kidney disease: A systematic review and meta-analysis

**DOI:** 10.1097/MD.0000000000050957

**Published:** 2026-10-02

**Authors:** Xiuzhen Shi, Kai Fu, Min Hu, Rui Li, Huimin Li, Shen Xu

**Affiliations:** a Department of Nephrology, General Hospital of the Yangtze River Shipping, Wuhan, Hubei, China.

**Keywords:** diabetic chronic kidney disease, Meta-analysis, Renal function, SGLT2 inhibitors

## Abstract

**Background:**

Chronic kidney disease (CKD) is a major global health burden characterized by progressive loss of renal function and high cardiovascular morbidity and mortality. Sodium-glucose cotransporter 2 (SGLT2) inhibitors, initially developed as glucose-lowering agents, have demonstrated pleiotropic metabolic and cardiorenal benefits. However, previous evidence syntheses have not fully clarified the magnitude of metabolic, hemodynamic, renal surrogate, and safety effects specifically in patients with diabetic CKD.

**Methods:**

A systematic search of PubMed, Embase, Web of Science, and the Cochrane Library was conducted up to December 2025. Randomized controlled trials (RCTs) evaluating SGLT2 inhibitors in patients with diabetic CKD were included. Outcomes were prespecified and grouped as primary renal surrogate marker outcomes (eGFR and UACR), secondary metabolic and hemodynamic outcomes (glycated hemoglobin, body weight, and blood pressure indicators), and safety outcomes (urinary tract infection and volume depletion-related adverse events). Meta-analyses were performed using RevMan 5.3 and R version 4.4.2 software.

**Results:**

A total of 17 randomized controlled trials involving 29,192 participants were included. For secondary metabolic and hemodynamic outcomes, SGLT2 inhibitors significantly reduced glycated hemoglobin levels (MD = −0.40%, 95% CI: −0.44 to −0.36), body weight (MD = −1.36 kg, 95% CI: −1.55 to −1.18), and systolic blood pressure (MD = −2.98 mm Hg, 95% CI: −3.44 to −2.52). For primary renal surrogate marker outcomes, no statistically significant differences were observed in eGFR (MD = 0.25 mL/min/1.73 m^2^, 95% CI: −0.08 to 0.57) or UACR (MD = −31.55 mg/g, 95% CI: −137.21 to 74.12). Regarding safety outcomes, SGLT2 inhibitors did not significantly increase the risk of urinary tract infections (RR = 1.20, 95% CI: 0.94 to 1.54) but were associated with a significantly higher risk of volume depletion-related adverse events (RR = 1.36, 95% CI: 1.16–1.59).

**Conclusion:**

In patients with diabetic CKD, SGLT2 inhibitors significantly improve glycemic control, reduce body weight, and lower systolic blood pressure, while showing neutral short- to medium-term effects on eGFR and albuminuria. These neutral surrogate-marker findings should be interpreted alongside large outcome trials showing long-term cardiorenal protection. Although SGLT2 inhibitors increase the risk of volume depletion-related adverse events, their overall safety profile remains acceptable with appropriate clinical monitoring.

## 1. Introduction

Chronic kidney disease (CKD) affects approximately 10% to 13% of the global population and is associated with substantial morbidity, mortality, and healthcare expenditure.^[1]^ Despite advances in blood pressure control and widespread use of renin–angiotensin system (RAS) blockade, a large proportion of patients continue to experience progressive renal function decline, ultimately leading to end-stage kidney disease (ESKD).^[2,3]^ For decades, therapeutic strategies capable of meaningfully modifying the natural course of CKD have remained limited.^[4]^

Sodium–glucose cotransporter 2 (SGLT2) inhibitors were initially developed as glucose-lowering agents for the treatment of type 2 diabetes mellitus (T2DM). However, accumulating evidence from large-scale cardiovascular and renal outcome trials has revealed that their protective effects extend far beyond glycemic control, encompassing improvements in renal and cardiovascular outcomes through multiple hemodynamic, metabolic, and anti-inflammatory mechanisms.^[5-7]^ Notably, these benefits have been consistently observed in patients both with and without diabetes, prompting a paradigm shift in contemporary CKD management.^[8]^

Recent landmark trials, including DAPA-CKD and EMPA-KIDNEY (The Study of Heart and Kidney Protection With Empagliflozin), have provided compelling evidence that SGLT2 inhibitors significantly reduce the risk of kidney failure, major cardiovascular events, and all-cause mortality in patients with CKD across a broad spectrum of baseline kidney function and albuminuria.^[8-10]^ Consequently, international clinical practice guidelines have now endorsed SGLT2 inhibitors as a foundational therapy for CKD.

However, several limitations of existing meta-analyses remain. First, some previous reviews pooled heterogeneous CKD populations irrespective of diabetes status, which may obscure drug effects in diabetic CKD, a population with distinct metabolic and hemodynamic features. Second, many analyses focused primarily on cardiovascular or hard renal composite outcomes and did not comprehensively quantify intermediate outcomes such as glycated hemoglobin (HbA1c), body weight (BW), blood pressure, estimated glomerular filtration rate (eGFR), and urinary albumin-to-creatinine ratio (UACR). Third, safety endpoints relevant to clinical use in CKD, particularly urinary tract infection and volume depletion-related adverse events (AEs), were not always evaluated in an integrated manner, and some prior analyses included relatively few randomized controlled trial (RCTs) from the diabetic CKD population. These evidence gaps highlight the need for a focused synthesis of efficacy and safety outcomes in patients with diabetic CKD.^[11,12]^

Therefore, this systematic review and meta-analysis aims to quantitatively evaluate the efficacy and safety of SGLT2 inhibitors in patients with diabetic CKD, with a particular focus on metabolic, hemodynamic, renal surrogate markers, and AEs, thereby providing clinically relevant evidence to inform real-world therapeutic decision-making and future translational research.

## 2. Materials and methods

### 2.1. Study design and literature search

This study was approved by the Ethics Committee of General Hospital of the Yangtze River Shipping. This systematic review and meta-analysis was conducted in accordance with the Preferred Reporting Items for Systematic Reviews and Meta-Analyses (PRISMA) guidelines. A comprehensive literature search was performed in PubMed, Embase, the Cochrane Library, and Web of Science from database inception to December 2025.

The search strategy combined Medical Subject Headings (MeSH) terms and free-text words related to sodium–glucose cotransporter 2 inhibitors and chronic kidney disease, including: “SGLT2 inhibitors,” “sodium-glucose cotransporter 2 inhibitors,” “canagliflozin,” “dapagliflozin,” “empagliflozin,” “chronic kidney disease,” and “diabetic kidney disease.” The detailed search strategies were adapted for each database.

### 2.2. Eligibility criteria

#### 2.2.1. Study design

Only randomized controlled trials (RCTs) published in English were eligible for inclusion.

#### 2.2.2. Participants

Studies enrolling adult patients (≥18 years) with diabetic chronic kidney disease were eligible. CKD was defined as an estimated glomerular filtration rate (eGFR) < 90 mL/min/1.73 m^2^ and/or evidence of kidney damage.

The exclusion criteria were as follows:

1. Nondiabetic CKD populations.
2. eGFR ≥ 90 mL/min/1.73 m^2^without evidence of kidney damage.
3. CKD requiring immunosuppressive therapy, dialysis, or kidney transplantation.
4. History of major cardiovascular or cerebrovascular events within the preceding 3 months.
5. Studies without a control group.
6. Prior treatment with SGLT2 inhibitors.
7. Duplicate publications, conference abstracts, case reports, reviews, and letters.

#### 2.2.3. Interventions and comparators

In the intervention group, patients received SGLT2 inhibitors in addition to standard care. The control group received a placebo or non–SGLT2 inhibitor glucose-lowering therapies plus standard care.

### 2.3. Outcome measures and endpoint classification

The primary outcomes were renal surrogate marker outcomes, defined as changes in estimated glomerular filtration rate (eGFR) and urinary albumin-to-creatinine ratio (UACR). These endpoints were selected because they reflect kidney function and albuminuria, which are commonly used to evaluate CKD progression in clinical trials.

Secondary efficacy outcomes were metabolic and hemodynamic outcomes, including changes in HbA1c, BW, and blood pressure indicators, particularly systolic blood pressure (SBP); diastolic blood pressure (DBP) was extracted when sufficient data were available.

Safety outcomes included the incidence of urinary tract infections (UTIs), volume depletion-related AEs (including hypotension, dehydration, or hypovolemia), and other reported AEs. All outcomes were analyzed by comparing the intervention and control groups.

### 2.4. Study selection, data extraction, and quality assessment

Two investigators independently screened titles and abstracts, reviewed the full texts, and determined study eligibility. Discrepancies were resolved through discussion or adjudication by a third reviewer.

Data were extracted independently using a predefined data extraction form, including study characteristics, patient demographics, intervention details, and outcome measures.

The methodological quality of the included studies was assessed using the Cochrane Risk of Bias tool, which evaluates the following domains: random sequence generation, allocation concealment, blinding, completeness of outcome data, selective reporting, and other potential sources of bias. Each domain was rated as having a low, high, or unclear risk of bias.^[13-29]^

### 2.5. Statistical analysis

Meta-analyses were performed using Review Manager (RevMan) version 5.3 and R version 4.4.2. Dichotomous outcomes were expressed as relative risks (RRs) with 95% confidence intervals (CIs), whereas continuous outcomes were expressed as mean differences (MDs) with 95% CIs.

When standard deviations (SDs) were not directly reported, they were calculated from standard errors (SEs), 95% CIs, or interquartile ranges (IQRs) according to established statistical methods.

Statistical heterogeneity was assessed using the Cochrane *Q* test and the *I*^2^ statistic. A fixed-effects model was applied when *I*^2^ ≤ 50%, and a random-effects model was used when *I*^2^ > 50%. Subgroup and sensitivity analyses were conducted to explore potential sources of heterogeneity. A 2-sided *P* value < .05 was considered statistically significant.

## 3. Results

### 3.1. Study selection and baseline characteristics

A comprehensive search of PubMed, Embase, Cochrane Library, and Web of Science initially identified 493 records. After removing 135 duplicates, 358 records underwent title/abstract screening, with 227 excluded. Full-text evaluation of the remaining 131 articles led to the exclusion of 114 (due to irrelevant outcomes, unavailable full text, or lack of data), leaving 17 publications (encompassing 31 randomized comparisons) that met inclusion criteria and were included in the meta-analysis. The SGLT2 inhibitors evaluated included canagliflozin, dapagliflozin, empagliflozin, luseogliflozin, ipragliflozin, sotagliflozin, and beigliflozin, with control groups receiving placebo. Follow-up durations were ≥ 24 weeks (3 studies exceeding 2 years). A total of 29,192 participants were included; the mean age across studies was > 55 years, and all had diabetic CKD. Detailed baseline characteristics are summarized in Table 1.

**Table 1:** Basic characters of the included studies.

Included study	Country/region	Study type	Intervention (experimental group/control group)	Sample size (experimental group/control group)	Outcome
Donald E. Kohan 2014^[13]^	Multinational	RCT	Dapagliflozin 10 mg/ Placebo	85/84	①②③④⑤
Masakazu Haneda 2016^[14]^	Japan	RCT	Luseogliflozin 2.5 mg/ Placebo	95/50	①②③④⑤
George Grunberger 2018^[15]^	Multinational	RCT	Empagliflozin 15 mg/ Placebo	155/154	①②③④⑥
Thea Anine 2019^[16]^	Norway	RCT	Empagliflozin 10 mg/ Placebo	22/22	①②④
Bruce Bode 2015^[17]^	Multinational	RCT	Canagliflozin 300 mg/ Placebo	236/237	①②③⑥
Jean-François Yale 2014^[18]^	Multinational	RCT	Canagliflozin 300 mg/ Placebo	89/90	①②③④⑥
Glenn M. 2022^[19]^	Multinational	RCT	Dapagliflozin 10 mg/ Placebo	451/437	⑥
Daisuke Yabe 2023^[20]^	Japan	RCT	Empagliflozin 10 mg/ Placebo	64/63	①②④
Atsunori Kashiwagi 2015^[21]^	Japan	RCT	Ipragliflozin 50 mg/ Placebo	118/46	①②④
Paola Fioretto 2018^[22]^	Multinational	RCT	Dapagliflozin 10 mg/ Placebo	160/161	①②③④⑤⑥
William G. 2023^[23]^	Multinational	RCT	Empagliflozin 10 mg/ Placebo	3304/3305	①②③⑤⑥
Wisanne M. 2025^[24]^	Multinational	RCT	Dapagliflozin 10 mg/ Placebo	750/750	⑤⑥
Claire C.J. 2018^[25]^	Multinational	RCT	Dapagliflozin 10 mg/ Placebo	93/69	①②③④⑤⑥
Hiddo J.L. 2020^[26]^	Multinational	RCT	Dapagliflozin 10 mg/ Placebo	2150/2150	③④⑤
Deepak L. 2021^[27]^	Multinational	RCT	Sotagliflozin (initial 200 mg/day, up-titrated to 400 mg/day if tolerated)/ Placebo	5292/5292	①②③④⑥
Andrew S. 2019^[28]^	Multinational	RCT	Beigliflozin 20 mg/ Placebo	157/155	①②③④⑤⑥
David C. 2021^[29]^	Multinational	RCT	Empagliflozin 10 mg/ Placebo	1455/1451	①④⑤

① HbA1c, ② BW, ③ SBP, ④ eGFR, ⑤ UACR, ⑥ AE (including urinary tract infections and volume depletion-related events, etc). All interventions were based on stable glycemic control and conventional CKD management.

AE = adverse event, BW = body weight, CKD = chronic kidney disease, eGFR = estimated glomerular filtration rate, HbA1c = glycated hemoglobin/ hemoglobin A1c, RCT = randomized controlled trial, SBP = systolic blood pressure, UACR = urinary albumin-to-creatinine ratio, UTI = urinary tract infection.

### 3.2. Risk of bias assessment

Risk of bias was assessed using the Cochrane Risk of Bias tool. Overall, most domains across the included RCTs were judged to be at low risk of bias, including random sequence generation, allocation concealment, and completeness of outcome data. Some studies were rated as having unclear risk in specific domains, primarily related to blinding or other potential sources of bias. No study was judged to be at high risk across multiple domains (Fig. 1).

**Figure 1.**
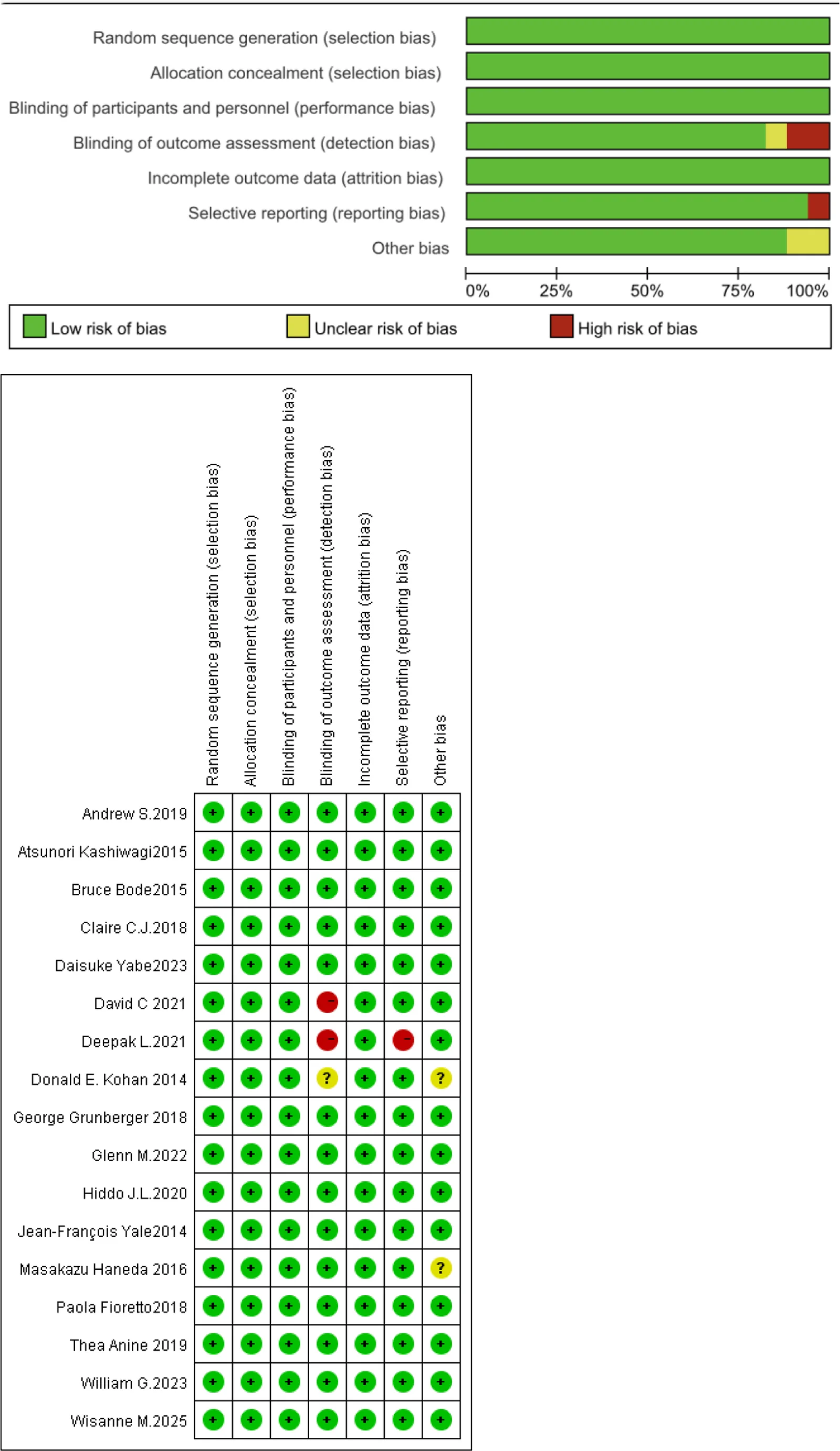
Inclusion of literature quality assessment.

### 3.3. Secondary metabolic and hemodynamic outcomes

#### 3.3.1. Glycated hemoglobin (HbA1c)

A total of 11 randomized controlled trials (enrolling 6452 patients in the experimental group and 6320 in the control group) were included to evaluate the effect of SGLT2 inhibitors on HbA1c in patients with diabetic CKD. Moderate-to-substantial heterogeneity was detected among the studies (*I*^2^ = 65.5%, *P* = .0012), and a random-effects model was applied for pooling.

The pooled analysis demonstrated that SGLT2 inhibitor therapy significantly reduced HbA1c levels compared with the control group, with a MD of −0.40 (95% CI: −0.44 to −0.36). The test for overall effect confirmed a statistically significant and robust glucose-lowering effect of SGLT2 inhibitors in patients with diabetic CKD (Fig. 2).

**Figure 2.**
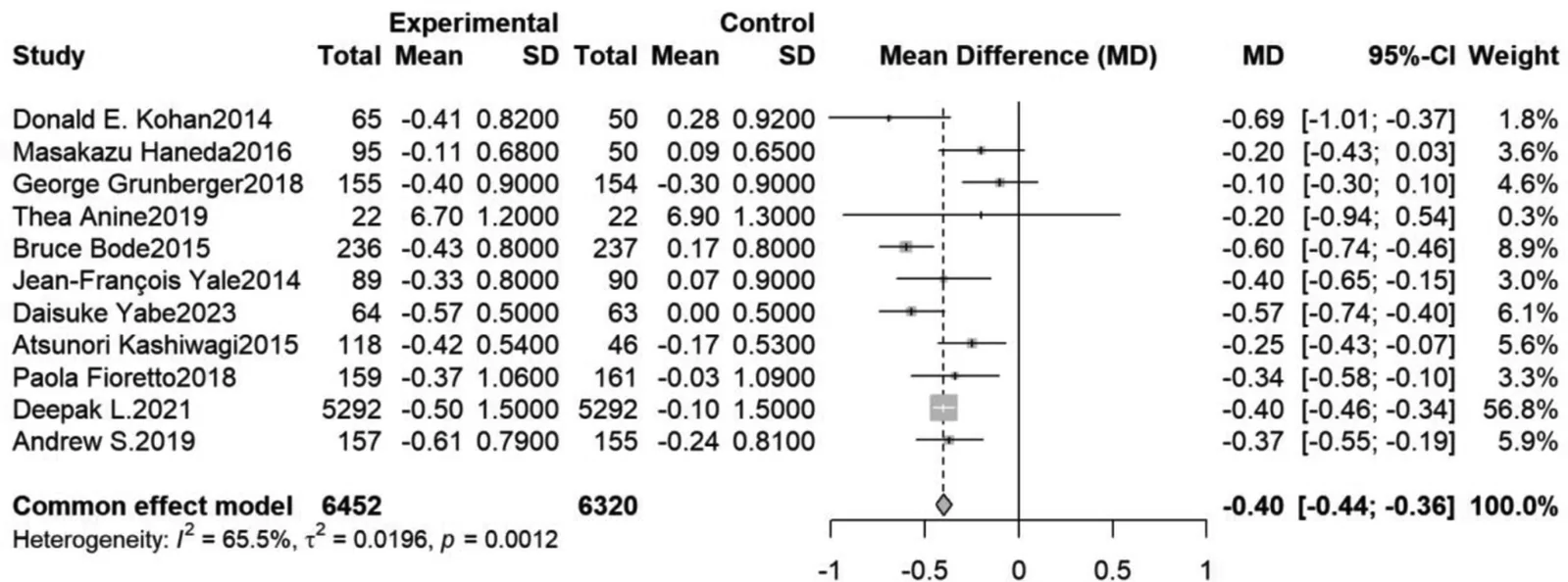
The effect of SGLT2 inhibitors on HbA1C in patients with diabetic chronic kidney disease. CI = confidence interval, HbA1c = glycated hemoglobin/ hemoglobin A1c, MD = mean difference, SD = standard deviation, SGLT2 = sodium–glucose cotransporter 2.

#### 3.3.2. Effects on body weight

To evaluate whether SGLT2 inhibitors reduce BW, we performed a meta-analysis of 12 randomized controlled trials, involving 9699 patients in the experimental group and 9554 in the control group. Heterogeneity assessment revealed moderate heterogeneity across the trials (*I*^2^ = 35.2%, τ^2^ = 0.2249, *P* = .1089). Since meta-regression did not identify significant sources of heterogeneity (including SGLT2 inhibitor type and dose), a common-effects model was applied for pooling. The results demonstrated that SGLT2 inhibitor therapy significantly reduced BW compared with the control group, with a MD of −1.36 (95% CI: −1.55 to −1.18). The test for overall effect yielded a robust weight-lowering effect of SGLT2 inhibitors (Fig. 3).

**Figure 3.**
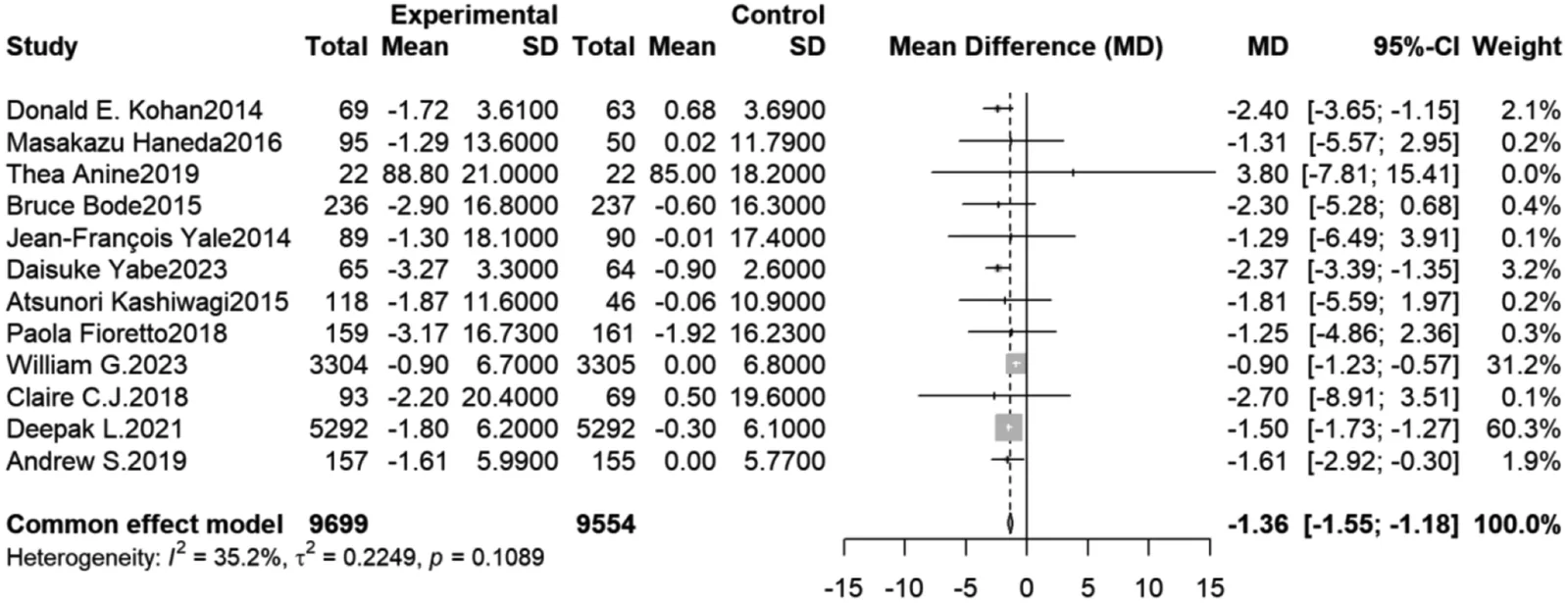
The effect of different types of SGLT2 inhibitors on BW in patients with diabetic chronic kidney disease. BW = body weight, CI = confidence interval, MD = mean difference, SD = standard deviation, SGLT2 = sodium–glucose cotransporter 2.

#### 3.3.3. Effects on SBP

Twelve studies reported changes in SBP. High heterogeneity was detected for SBP analyses (*I*^2^ = 70.8%). Pooled results demonstrated that SGLT2 inhibitors significantly reduced SBP (MD = −2.98 mm Hg, 95% CI −3.44 to −2.52; *P* < .001; Fig. 4).

**Figure 4.**
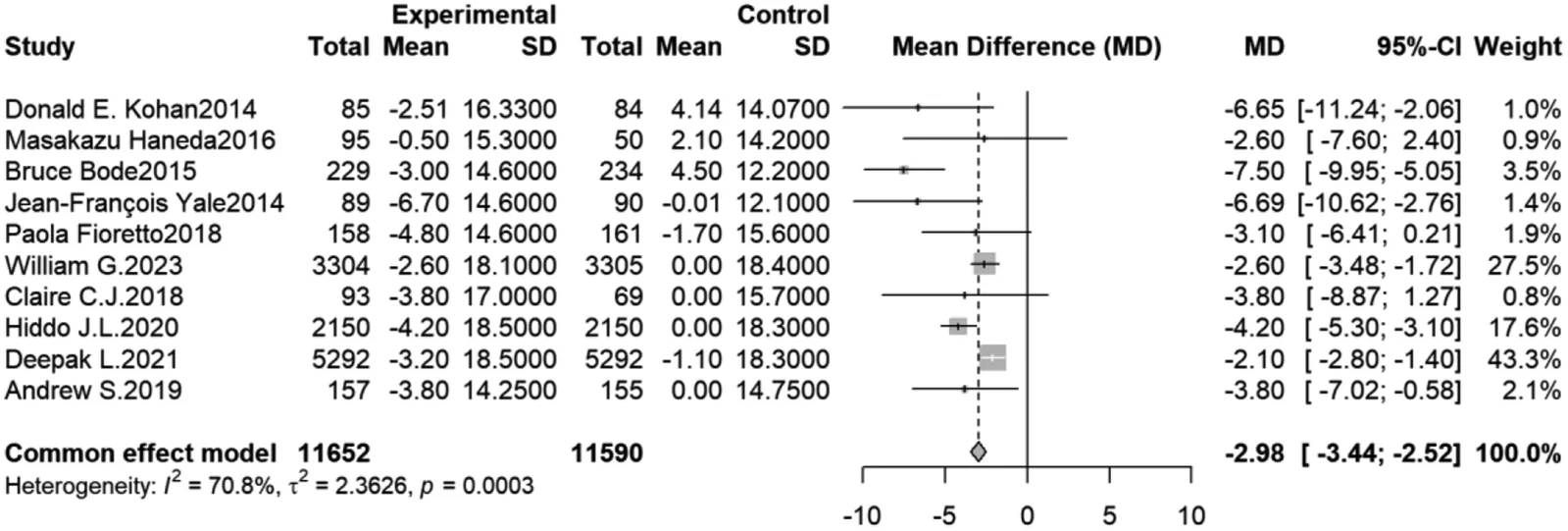
The effect of SGLT2 inhibitors on SBP in patients with diabetic chronic kidney disease. CI = confidence interval, MD = mean difference, SBP = systolic blood pressure, SD = standard deviation, SGLT2 = sodium–glucose cotransporter 2.

### 3.4. Primary renal surrogate marker outcomes

#### 3.4.1. Effects on eGFR

To assess the effect of SGLT2 inhibitors on eGFR in patients with diabetic CKD, we included 14 randomized controlled trials (enrolling 12,502 patients in the experimental group and 12,342 in the control group). Heterogeneity evaluation revealed substantial heterogeneity across the studies (*I*^2^ = 77.2%, τ^2^ = 2.7486, *P* < .0001). Thus, a random-effects model was employed for data pooling. The pooled results demonstrated that SGLT2 inhibitor therapy did not yield a statistically significant change in eGFR compared with the control group, with a mean difference (MD) of 0.25 mL/min/1.73 m^2^ (95% CI: −0.08 to 0.57; *P* = .14; Fig. 5).

**Figure 5.**
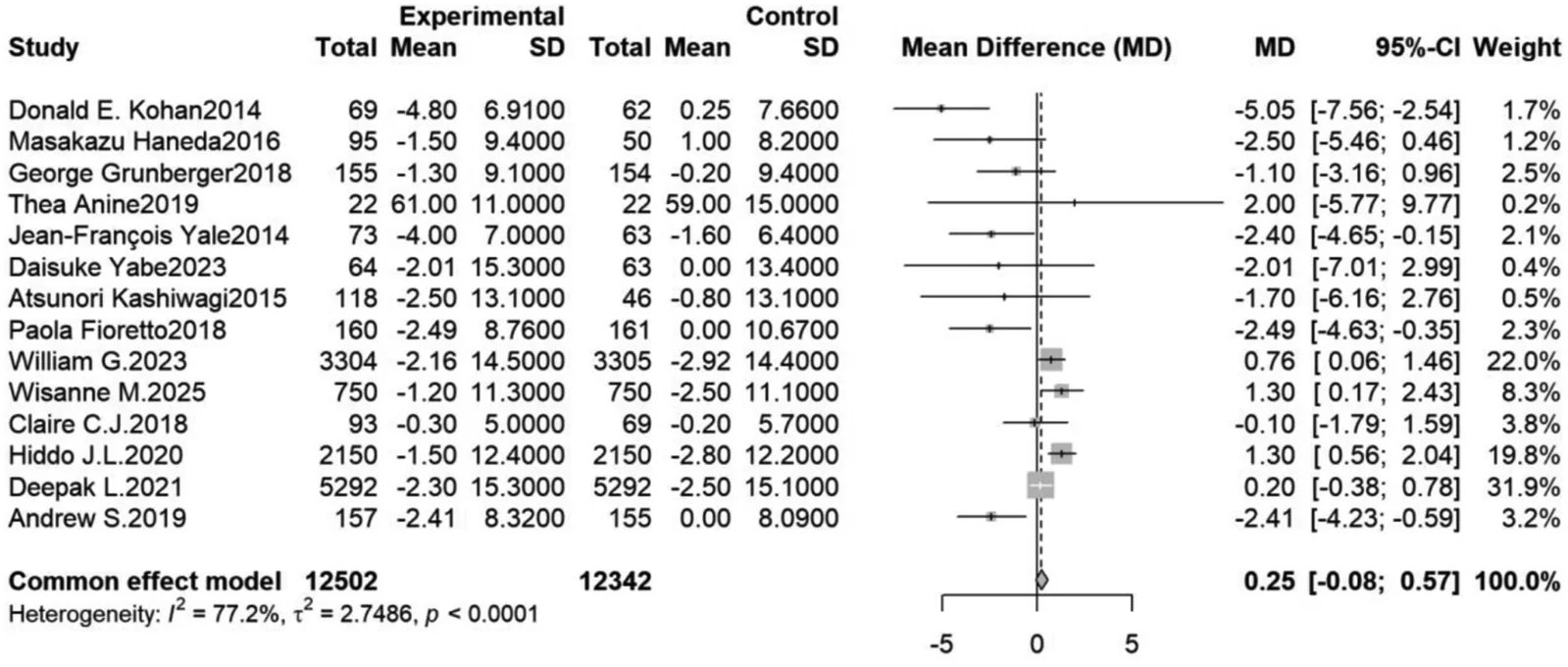
The effect of SGLT2 inhibitors in different subgroups of eGFR on SBP in patients with diabetic chronic kidney disease. CI = confidence interval, eGFR = estimated glomerular filtration rate, MD = mean difference, SBP = systolic blood pressure, SD = standard deviation, SGLT2 = sodium–glucose cotransporter 2.

#### 3.4.2. Effects on UACR

To evaluate SGLT2 inhibitors’ effect on the UACR in diabetic CKD patients, 3 randomized controlled trials (332 vs 283 patients in experimental vs control groups) were included. No heterogeneity was observed (*I*^2^ = 0.0%, *P* = .7099), so a fixed-effects model was used. Pooled results showed no statistically significant difference in UACR between groups (MD = −31.55, 95% CI [−137.21, 74.12], *P* = .58; Fig. 6).

**Figure 6.**
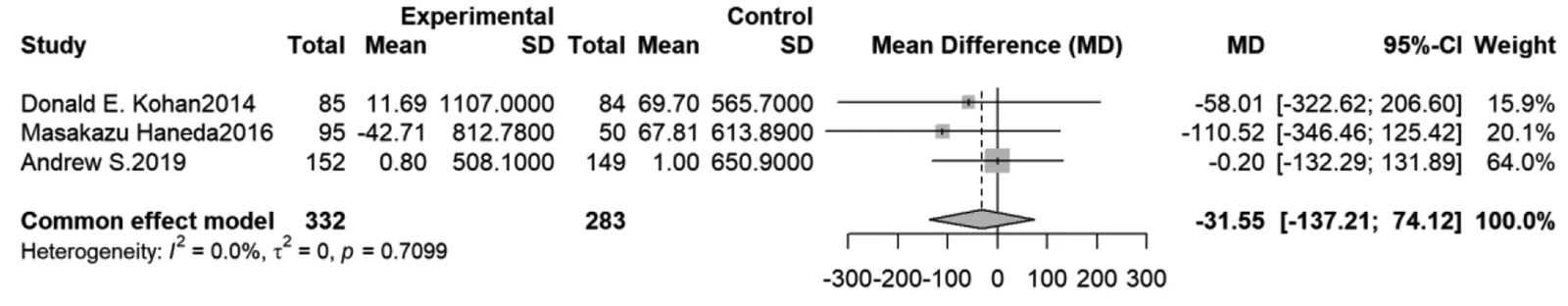
The effect of SGLT2 inhibitors on UACR in patients with diabetic chronic kidney disease. CI = confidence interval, MD = mean difference, SD = standard deviation, SGLT2 = sodium–glucose cotransporter 2, UACR = urinary albumin-to-creatinine ratio.

### 3.5. Safety outcomes

#### 3.5.1. Urinary tract infection adverse events

To assess the risk of UTIs with SGLT2 inhibitors in patients with diabetic CKD, 8 randomized controlled trials (3129 vs. 3023 patients in experimental vs. control groups) were included. Low heterogeneity was observed (*I*^2^ = 15.3%, *P* = .3098), so a fixed-effects model was applied. Pooled results showed no statistically significant difference in UTI risk between the SGLT2 inhibitor group and the control group (risk ratio [RR] = 1.20, 95% confidence interval [CI]: 0.94–1.54, *P* = .14; Fig. 7).

**Figure 7.**
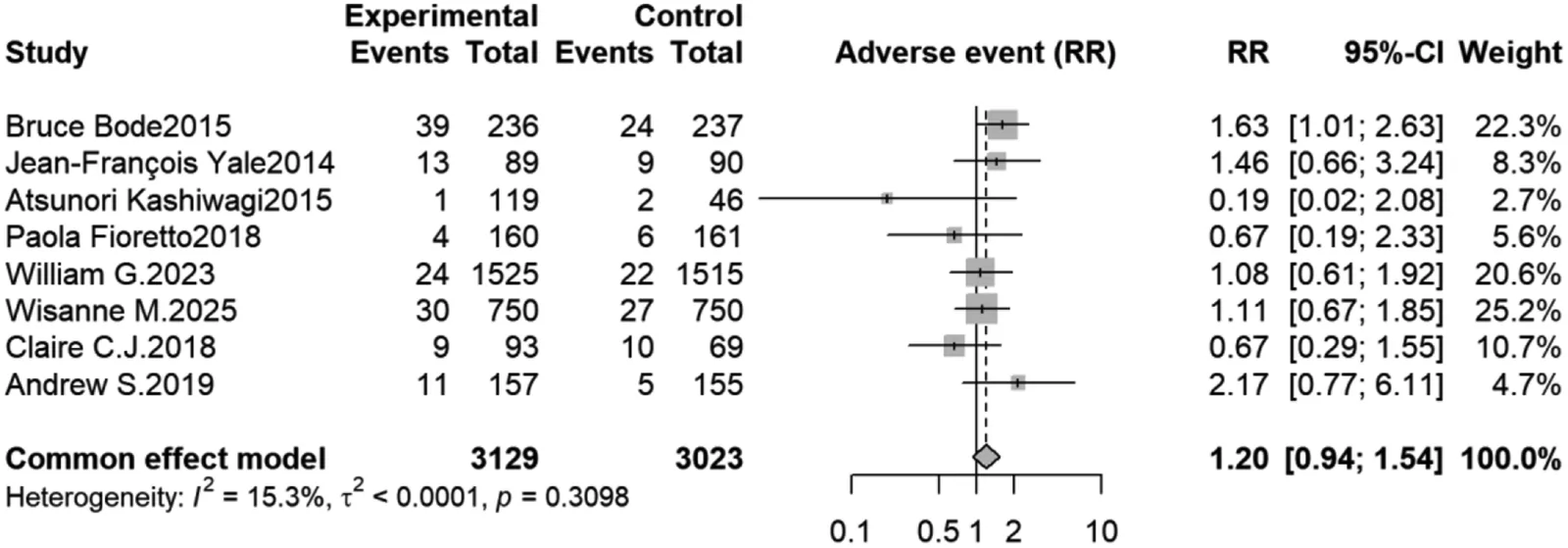
The effect of SGLT2 inhibitors on UTI in patients with diabetic chronic kidney disease. CI = confidence interval, RR = risk ratio, SGLT2 = sodium–glucose cotransporter 2, UTI = urinary tract infection.

#### 3.5.2. Volume depletion-related adverse events

For volume depletion-related AEs, 6 randomized controlled trials (6387 vs. 6364 patients in experimental vs. control groups) were included. Low heterogeneity was detected (*I*^2^ = 13.1%, *P* = .3307), and a fixed-effects model was used. Pooled analysis revealed a statistically significant increased risk of volume depletion in the SGLT2 inhibitor group compared with the control group (RR = 1.36, 95% CI: 1.16–1.59, *P* < .001; Fig. 8).

**Figure 8.**
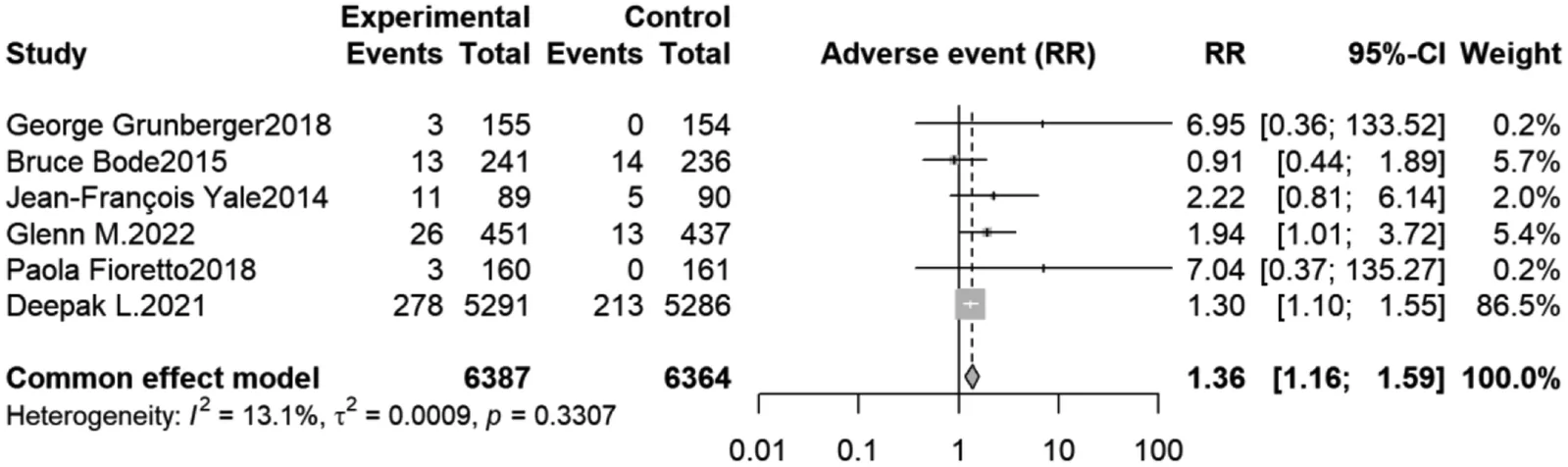
The effect of SGLT2 inhibitors on volume depletion-related in patients with diabetic chronic kidney disease. CI = confidence interval, RR = risk ratio, SGLT2 = sodium–glucose cotransporter 2.

## 4. Discussion

In this systematic review and meta-analysis of 17 randomized controlled trials involving 29,192 participants, we comprehensively evaluated SGLT2 inhibitors in patients with diabetic chronic kidney disease (DKD) according to 3 prespecified outcome domains: primary renal surrogate marker outcomes, secondary metabolic and hemodynamic outcomes, and safety outcomes. SGLT2 inhibitors significantly improved glycemic control, reduced BW, and lowered systolic blood pressure, while showing neutral effects on eGFR and UACR over the study durations analyzed. From a safety perspective, SGLT2 inhibitors did not significantly increase the risk of urinary tract infections but were associated with a higher risk of volume depletion-related AEs.

### 4.1. Metabolic and hemodynamic effects

Our meta-analysis showed that SGLT2 inhibitors significantly reduced HbA1c (MD = −0.40%), BW (MD = −1.36 kg), and systolic blood pressure (MD = −2.98 mm Hg). These findings are fully consistent with the established pharmacological profile of SGLT2 inhibitors and confirm that their metabolic and hemodynamic benefits are preserved in patients with DKD. Importantly, these effects are clinically relevant, as hyperglycemia, obesity, and hypertension are well-recognized drivers of CKD progression and cardiovascular risk.^[30]^

The concomitant reductions in BW and blood pressure are largely attributable to mild osmotic diuresis, natriuresis, and negative energy balance induced by glycosuria. Unlike many conventional antihypertensive or glucose-lowering agents, SGLT2 inhibitors reduce blood pressure without activating the sympathetic nervous system and achieve weight loss without promoting fluid retention, making them particularly attractive for patients with CKD.^[31,32]^

### 4.2. Effects on renal surrogate markers

A notable finding of our analysis is that SGLT2 inhibitors did not produce a statistically significant short- to medium-term change in eGFR (MD = 0.25 mL/min/1.73 m^2^) or UACR (MD = −31.55 mg/g) compared with control treatment. This neutral result should not be interpreted as an absence of renoprotection. Instead, it likely reflects the timing and type of renal endpoints available for pooling. SGLT2 inhibitors characteristically produce a biphasic eGFR response, with an early, reversible hemodynamic dip followed by slower chronic eGFR decline during longer follow-up. Mechanistically, SGLT2 inhibition reduces proximal tubular sodium reabsorption, increases sodium delivery to the macula densa, and restores tubuloglomerular feedback, resulting in afferent arteriolar vasoconstriction and reduced intraglomerular pressure.^[33]^ This hemodynamic adjustment leads to an early, reversible reduction in eGFR that reflects functional rather than structural renal changes. Over longer time horizons, this reduction in intraglomerular pressure is believed to protect the glomerulus from hyperfiltration-induced injury and slow structural disease progression.^[34]^

The neutral UACR result in the present meta-analysis may be explained by the small number of included UACR studies, substantial variability in baseline albuminuria, and relatively short follow-up. Importantly, large outcome-driven trials provide complementary evidence. CREDENCE demonstrated that canagliflozin reduced kidney failure and cardiovascular events in patients with type 2 diabetes and kidney disease, while DAPA-CKD and EMPA-KIDNEY showed that dapagliflozin and empagliflozin reduced risks of CKD progression and cardiorenal composite outcomes across broad CKD populations, including patients with diabetes. Therefore, our results should be viewed as a pooled analysis of short- to medium-term surrogate marker changes, whereas large trials support the long-term kidney-protective effect of SGLT2 inhibitors on hard renal outcomes. Future analyses should further evaluate chronic eGFR slope, sustained eGFR decline, kidney failure, and albuminuria response according to baseline eGFR, albuminuria category, diabetes status, and treatment duration.

### 4.3. Biological plausibility and mechanistic insights

Beyond their hemodynamic actions, SGLT2 inhibitors exert multiple complementary renoprotective effects that are largely independent of glucose lowering. By decreasing sodium and glucose reabsorption in the proximal tubule, these agents reduce tubular workload and oxygen consumption, thereby improving cortical oxygenation and mitigating hypoxic tubular injury. Experimental and clinical studies further suggest that SGLT2 inhibitors attenuate oxidative stress, suppress inflammatory cytokine production, and inhibit profibrotic signaling pathways, contributing to reduced tubulointerstitial fibrosis and structural kidney damage.^[35,36]^

In addition, SGLT2 inhibitors induce mild and sustained natriuresis and osmotic diuresis, leading to reductions in plasma volume and blood pressure, while favorably modulating the renin–angiotensin–aldosterone system and improving endothelial function.^[37]^ These pleiotropic effects provide a coherent mechanistic framework explaining the broad renal and cardiovascular benefits observed in large-scale outcome trials.^[8]^

### 4.4. Safety considerations

With respect to safety, our meta-analysis found no statistically significant increase in the risk of urinary tract infections associated with SGLT2 inhibitor therapy, which is reassuring given longstanding concerns regarding genitourinary infections. However, we did observe a significantly higher risk of volume depletion–related AEs, consistent with the diuretic and natriuretic properties of this drug class. Clinicians should therefore pay particular attention to volume status, especially in elderly patients, those receiving concomitant diuretics, or individuals with advanced CKD. Overall, the safety profile observed in our analysis supports the continued and cautious use of SGLT2 inhibitors in DKD, provided that appropriate patient selection and clinical monitoring are implemented.

### 4.5. Limitations

Several limitations of this meta-analysis should be acknowledged. First, although this study focused specifically on diabetic CKD, the follow-up duration of most included trials ranged from 24 weeks to 2 years, which may be insufficient to capture long-term changes in chronic eGFR slope, albuminuria trajectory, kidney failure, or sustained renal composite outcomes. Second, heterogeneity was moderate to substantial for some outcomes, likely reflecting differences in study populations, drug types, dosages, baseline renal function, albuminuria categories, and concomitant therapies. Third, the number of studies reporting UACR was relatively small, limiting the statistical power for this endpoint and increasing uncertainty around the pooled estimate. Fourth, hard renal outcomes and individual patient-level data were unavailable for unified pooling, precluding more refined subgroup, time-to-event, and interaction analyses. These limitations should be considered when interpreting the neutral eGFR and UACR findings.

## 5. Conclusion

In conclusion, this meta-analysis demonstrates that SGLT2 inhibitors significantly improve glycemic control, reduce BW, and lower systolic blood pressure in patients with diabetic CKD, while exerting neutral effects on short- to medium-term eGFR and albuminuria and maintaining an acceptable safety profile, aside from an increased risk of volume depletion. The neutral renal surrogate marker results are biologically plausible in light of the early hemodynamic eGFR dip and limited UACR data, and they should be interpreted together with large outcome trials demonstrating long-term kidney protection. These findings further support the integration of SGLT2 inhibitors into the comprehensive management of DKD and underscore their role as a cornerstone therapy in modern nephrology.

## Acknowledgments

The author wishes to express their gratitude to the team for their valuable contributions, help, and insightful discussions throughout the project.

## Author contributions

**Conceptualization:** Xiuzhen Shi, Kai Fu, Min Hu, Rui Li, Huimin Li, Shen Xu.

**Data curation:** Xiuzhen Shi, Kai Fu, Min Hu, Rui Li, Huimin Li, Shen Xu.

**Formal analysis:** Xiuzhen Shi, Kai Fu, Min Hu, Rui Li, Huimin Li, Shen Xu.

**Funding acquisition:** Xiuzhen Shi, Kai Fu, Min Hu, Rui Li, Huimin Li, Shen Xu.

**Investigation:** Xiuzhen Shi, Kai Fu, Shen Xu.

**Writing – original draft:** Shen Xu.

**Writing – review & editing:** Shen Xu.
